# Supplementary material for: Host proteostasis modulates influenza evolution
Source: eLife. 2017 Sep 26;6:e28652. doi: 10.7554/eLife.28652 (PMC5614556; doi:10.7554/eLife.28652)
Supplement: Figure 4—source data 1. [file elife-28652-fig4-data1.pdf]

Figure 4–source data 1. List of ten highest % frequency non-synonymous mutations for each proteostasis environment and passage.

**HSF1-Activated**									
**Passage 2**		**Passage 9**		**Passage 11**		**Passage 19**		**Passage 23**	
Mutation	Freq.	Mutation	Freq.	Mutation	Freq.	Mutation	Freq.	Mutation	Freq.
**NS1 F150S**	**41**	**HA N144T**	**88**	**HA N144T**	**98**	**HA M246I**	**100**	**HA M246I**	**100**
**HA N144T**	**30**	**HA V242I**	**87**	**HA V242I**	**97**	**HA V242I**	**100**	**HA V242I**	**100**
**PA H452Q**	**25**	**HA M246I**	**86**	**HA M246I**	**97**	**HA N144T**	**100**	**HA N144T**	**100**
**HA M246I**	**24**	PB2 F14S	76	*PB2 F14S*	*96*	**PB2 F14S**	**100**	**PB2 F14S**	**100**
**HA N262K**	**17**	**NS1 F150S**	**37**	**PA H452Q**	**26**	**NP G384R**	**94**	*NP G384R*	*100*
**HA V242I**	**11**	**PA H452Q**	**34**	**NS1 F150S**	**26**	NP D101G	55	NP D101G	91
HA N142K	*4*	PA V44A	16	*NP G384R*	*23*	M1 K174R	43	M1 K174R	89
HA E402G	*4*	*NP G384R*	*13*	PB1 Y49*	15	PB1 I517V	41	PB1 Y49*	64
NS1 R37G	3	PB2 E2K	12	PA V44A	15	PB1 Y49*	33	PB1 I517V	60
NS1 N127T	2	**PB1 E697K**	**10**	NP D480N	11	PB1 H456N	26	PA E604G	26

**Baseline**									
**Passage 2**		**Passage 9**		**Passage 11**		**Passage 19**		**Passage 23**	
Mutation	Freq.	Mutation	Freq.	Mutation	Freq.	Mutation	Freq.	Mutation	Freq.
**NS1 F150S**	**36**	**HA N144T**	**73**	**HA N144T**	**78**	**HA V242I**	**100**	**HA V242I**	**100**
**PA H452Q**	**34**	**HA V242I**	**72**	**HA V242I**	**78**	**HA M246I**	**100**	**HA M246I**	**100**
**HA N144T**	**26**	**HA M246I**	**72**	**HA M246I**	**77**	**HA N144T**	**100**	**PB2 F14S**	**100**
**HA M246I**	**21**	**PA H452Q**	**49**	**PA H452Q**	**54**	*PB2 F14S*	*100*	**HA N144T**	**100**
**HA N262K**	**20**	PB1 V660F	46	PB1 V660F	51	NP D101N	80	NP G384R	94
**HA N142K**	**7**	PA N359T	22	PA N359T	30	HA I538T	78	PA M12I	93
NS1 R118K	6	**NS1 F150S**	**17**	NP D101N	26	PA M12I	63	NP D101N	88
PB1-F2 R21K	4	**PB1 F700S**	**15**	PB2 F14S	22	*PA H452Q*	*60*	HA I538T	80
HA G140D	4	NP G384R	13	NS1 R118K	22	HA G349S	57	PA E15K	64
NP V100I	3	NP D101N	13	**PB1 F700S**	**18**	NP G384R	54	*PA H452Q*	*63*

**Hsp90-Inhibited**									
**Passage 2**		**Passage 9**		**Passage 11**		**Passage 19**		**Passage 23**	
Mutation	Freq.	Mutation	Freq.	Mutation	Freq.	Mutation	Freq.	Mutation	Freq.
**HA N144T**	**32**	*HA N144T*	*53*	*HA N144T*	*70*	*HA M246I*	*100*	HA M246I	100
**PA H452Q**	**28**	HA V242I	49	*HA V242I*	*69*	*HA V242I*	*100*	HA N144T	100
**NS1 F150S**	**25**	*HA M246I*	*48*	*HA M246I*	*68*	*HA N144T*	*100*	HA V242I	100
**HA M246I**	**19**	**PA H452Q**	**41**	**PA H452Q**	**46**	HA A492V	99	*M1 K174R*	*100*
**HA N262K**	**18**	NP N319K	28	NP N319K	39	HA Y249H	98	HA Y249H	99
**HA N142K**	**12**	NS1 R118K	18	HA Y249H	35	*PB2 F14S*	*95*	HA A492V	99

NS1 I182T; NEP S27P	9	NS1 I182T; NEP S27P	18	NS1 R118K	26	M1 K174R	86	*PB2 F14S*	*98*
NS1 R118K	9	HA Y249H	15	NS1 I182T; NEP S27P	26	*NP N319K*	*81*	*NP N319K*	*96*
HA E402G	*5*	PA D3Y	11	HA A492V	19	**PA H452Q**	**71**	**PA H452Q**	**93**
M2 A30T	4	NS1/NEP V6L	11	NS1/NEP V6L	17	*M1 T239A*	*61*	HA R241G	83

*Bolded mutations were observed in 3/3 biological replicates; italicized mutations were observed in 2/3 biological replicates; codon number is in parentheses.
