# Supplementary material for: Host proteostasis modulates influenza evolution
Source: eLife. 2017 Sep 26;6:e28652. doi: 10.7554/eLife.28652 (PMC5614556; doi:10.7554/eLife.28652)
Supplement: Figure 4—source data 2. [file elife-28652-fig4-data2.pdf]

**Figure 4–source data 2.** List of ten highest % frequency synonymous mutations for each proteostasis environment and passage.

| **HSF1-Activated** | | | | | | | | | |
|---|---|---|---|---|---|---|---|---|---|
| **Passage 2** | | **Passage 9** | | **Passage 11** | | **Passage 19** | | **Passage 23** | |
| Mutation | Freq. | Mutation | Freq. | Mutation | Freq. | Mutation | Freq. | Mutation | Freq. |
| **6555 A/G (Intergenic)** | **42** | **6555 A/G (Intergenic)** | **42** | **6555 A/G (Intergenic)** | **43** | 7689 ggT/ggC (PA 371) | 78 | 6414 aaA/aaG (NP 452) | 92 |
| 962 atC/atT (HA 316) | 8 | 5202 aaA/aaG (NP 48) | 13 | 5202 aaA/aaG (NP 48) | 18 | 6414 aaA/aaG (NP 452) | 60 | 7689 ggT/ggC (PA 371) | 83 |
| 4905 caC/caT (NEP 87) | 5 | 1922 ttG/ttA (M1 60) | 10 | *7770 ccT/ccG (PA 398)* | *16* | **6555 A/G (Intergenic)** | **43** | **6555 A/G (Intergenic)** | **42** |
| *7647 acT/acA (PA 357)* | *2* | *2586 taT/taC (M2 52)* | *10* | 1922 ttG/ttA (M1 60) | 15 | *1238 ttC/ttT (HA 408)* | *35* | 1238 ttC/ttT (HA 408) | 30 |
| 5109 ggG/ggA (NP 17) | 2 | **6687 gcA/gcG (PA 37)** | **10** | *2586 taT/taC (M2 52)* | *14* | 2586 taT/taC (M2 52) | 34 | **10867 ttT/ttC (PB1 700)** | **20** |
| 8607 gaA/gaG (PA 677) | 2 | 2411 ccT/ccC (M1 223) | 9 | **6687 gcA/gcG (PA 37)** | **13** | 9292 gaT/gaC (PB1 175) | 34 | 9292 gaT/gaC (PB1 175) | 20 |
| 9131 Cta/Tta (PB1 122) | 2 | 962 atC/atT (HA 316) | 7 | 7689 ggT/ggC (PA 371) | 12 | 9229 ggT/ggC (PB1 154) | 19 | 11135 gaG/gaA (PB2 18) | 18 |
| 10249 agC/agT (PB1 494) | 2 | 7869 gaC/gaT (PA 431) | 7 | 8721 ttA/ttG (PA 715) | 11 | 5202 aaA/aaG (NP 48) | 16 | 6696 acT/acA (PA 40) | 17 |
| | | 8166 ccG/ccA (PA 530) | 7 | 4223 ctT/ctC (NS1 82) | 10 | 7980 gcC/gcT (PA 468) | 12 | 9229 ggT/ggC (PB1 154) | 17 |
| | | 7689 ggT/ggC (PA 371) | 6 | 7869 gaC/gaT (PA 431) | 9 | 4223 ctT/ctC (NS1 82) | 11 | *8529 gcA/gcC (PA 651)* | *10* |

| **Baseline** | | | | | | | | | |
|---|---|---|---|---|---|---|---|---|---|
| **Passage 2** | | **Passage 9** | | **Passage 11** | | **Passage 19** | | **Passage 23** | |
| Mutation | Freq. | Mutation | Freq. | Mutation | Freq. | Mutation | Freq. | Mutation | Freq. |
| **6555 A/G (Intergenic)** | **43** | **6555 A/G (Intergenic)** | **43** | **6555 A/G (Intergenic)** | **43** | 2586 taT/taC (M2 52) | 67 | *11135 gaG/gaA (PB2 18)* | *95* |
| *4905 caC/caT (NEP 87)* | *14* | 10960 gaG/gaA (PB1 731) | 25 | *11135 gaG/gaA (PB2 18)* | 34 | *11135 gaG/gaA (PB2 18)* | *63* | 2586 taT/taC (M2 52) | 68 |
| *4346 gcA/gcG (NS1 56)* | 11 | 4905 caC/caT (NEP 87) | 20 | 4905 caC/caT (NEP 87) | 24 | 2093 ctC/ctA (M1 117) | 60 | 2093 ctC/ctA (M1 117) | 63 |
| 8217 gaT/gaC (PA 547) | 5 | 4346 gcA/gcG (NS1 56) | 16 | 4346 gcA/gcG (NS1 56) | 20 | **6555 A/G (Intergenic)** | **44** | **6555 A/G (Intergenic)** | **43** |
| 6207 agC/agT (NP 383) | 4 | 11109 Ctg/Ttg (PB2 10) | 14 | 10960 gaG/gaA (PB1 731) | 20 | 10444 taT/taC (PB1 559) | 21 | 10444 taT/taC (PB1 559) | 42 |
| 10024 ttA/ttG (PB1 414) | 2 | 6045 gtG/gtA (NP 329) | 12 | 2586 taT/taC (M2 52) | 16 | 2605 Ctg/Ttg (M2 59) | 20 | 5175 ttC/ttT (NP 39) | 26 |
| 4409 ctT/ctC (NS1 77) | 2 | 6207 agC/agT (NP 383) | 9 | 8013 gaT/gaC (PA 479) | 12 | 4905 caC/caT (NEP 87) | 18 | 4905 caC/caT (NEP 87) | 23 |
| 779 ccG/ccA (HA 255) | 2 | 8013 gaT/gaC (PA 479) | 8 | 11109 Ctg/Ttg (PB2 10) | 11 | 4346 gcA/gcG (NS1 56) | 15 | 6630 gaA/gaG (PA 18) | 22 |
| **7647 acT/acA (PA 357)** | **2** | 8217 gaT/gaC (PA 547) | 8 | 8217 gaT/gaC (PA 547) | 9 | 7662 aaA/aaG (PA 362) | 13 | 4346 gcA/gcG (NS1 56) | 21 |

| | | | | | | | | | |
|---|---|---|---|---|---|---|---|---|---|
| 10351 acA/acG (PB1 528) | 2 | 425 acC/acT (HA 137) | 7 | 2093 ctC/ctA (M1 117) | 9 | 8685 aaT/aaC (PA 703) | 13 | 2605 Ctg/Ttg (M2 59) | 19 |
| **Hsp90-Inhibited** | | | | | | | | | |
| **Passage 2** | | **Passage 9** | | **Passage 11** | | **Passage 19** | | **Passage 23** | |
| Mutation | Freq. | Mutation | Freq. | Mutation | Freq. | Mutation | Freq. | Mutation | Freq. |
| **6555 A/G (Intergenic)** | **42** | **6555 A/G (Intergenic)** | **43** | *2616 ggG/ggA (M2 62)* | *51* | 11135 gaG/gaA (PB2 18) | 92 | 11135 gaG/gaA (PB2 18) | 94 |
| 2616 ggG/ggA (M2 62) | 28 | 2616 ggG/ggA (M2 62) | 38 | **6555 A/G (Intergenic)** | **43** | *2586 taT/taC (M2 52)* | *83* | 5592 gcT/gcC (NP 178) | 93 |
| 2718 ctG/ctA (M2 96) | 4 | 2270 gaG/gaA (M1 176) | 34 | 2270 gaG/gaA (M1 176) | 37 | 2616 ggG/ggA (M2 62) | 79 | 2616 ggG/ggA (M2 62) | 90 |
| 2297 acG/acA (M1 185) | 3 | 4905 caC/caT (NEP 87) | 23 | 5592 gcT/gcC (NP 178) | 24 | 5592 gcT/gcC (NP 178) | 61 | 1001 acT/acC (HA 329) | 58 |
| 4361 ggA/ggG (NS1 61) | 2 | 5592 gcT/gcC (NP 178) | 11 | 4905 caC/caT (NEP 87) | 22 | 4905 caC/caT (NEP 87) | 45 | 4905 caC/caT (NEP 87) | 56 |
| 11135 gaG/gaA (PB2 18) | 2 | 9220 agA/agG (PB1 151) | 10 | 2586 taT/taC (M2 52) | 17 | **6555 A/G (Intergenic)** | **42** | 10309 gtG/gtA (PB1 514) | 51 |
| 4905 caC/caT (NEP 87) | 2 | 2586 taT/taC (M2 52) | 7 | 11135 gaG/gaA (PB2 18) | 17 | 1001 acT/acC (HA 329) | 36 | 2586 taT/taC (M2 52) | 44 |
| *7647 acT/acA (PA 357)* | 2 | 5784 gtG/gtA (NP 242) | 4 | 1694 atT/atC (HA 560) | 9 | 2099 taT/taC (M1 119) | 33 | 5214 taT/taC (NP 52) | 43 |
| **10249 agC/agT (PB1 494)** | **2** | 7902 agG/agA (PA 442) | 3 | 9220 agA/agG (PB1 151) | 7 | 5826 ctC/ctA (NP 256) | 19 | **6555 A/G (Intergenic)** | **42** |
| | | 7512 aaA/aaG (PA 312) | 3 | 4436 gcT/gcA (NS1 86) | 4 | 10309 gtG/gtA (PB1 514) | 16 | 2099 taT/taC (M1 119) | 22 |

*Bolded mutations were observed in 3/3 biological replicates; italicized mutations were observed in 2/3 biological replicates. Top line of mutation description is the nucleotide number (of concatenated influenza genome) and codon mutation; bottom line contains the respective protein and codon number in parentheses. Empty cells indicate that less than ten variants were observed.
