# Supplementary material for: Host proteostasis modulates influenza evolution
Source: eLife. 2017 Sep 26;6:e28652. doi: 10.7554/eLife.28652 (PMC5614556; doi:10.7554/eLife.28652)
Supplement: Figure 5—source data 1. — *Excludes contribution from solute configuration entropy. [file elife-28652-fig5-data1.pdf]

Figure 5–source data 1. Molecular dynamics simulations: Energy contributions

PA:PB1 Variant(s)										
**Contribution (kcal/mol)**	**WT:WT**	**W619R:WT**	**P620A:WT**	**P620T:WT**	**P620S:WT**	**E629K:WT**	**WT:K11E**	**WT:V12D**	**E629K:K11E**	**E629K:V12D**
E_{ele}	−183.7±15.1	−219.9±16.8	−269.7±16.2	−150.8±10.2	−304.0±18.0	−201.1±15.7	158.2±23.7	−155.9±12.3	29.9±8.0	−85.6±11.2
E_{vdw}	−119.5±2.2	−129.9±2.1	−120.8±1.0	−121.1±1.1	−115.2±1.4	−136.0±1.5	−116.6±1.5	−122.2±0.9	−129.5±0.3	−131.7±1.4
E_{int}	0.0±0.0	0.0±0.0	0.0±0.0	0.0±0.0	0.0±0.0	0.0±0.0	0.0±0.0	0.0±0.0	0.0±0.0	0.0±0.0
$E_{gas} = E_{ele} + E_{vdw} + E_{int}$	−303.2±16.2	−349.8±18.8	−390.5±16.5	−271.9±10.2	−419.2±16.8	−337.2±15.9	41.5±24.9	−278.1±12.9	−99.6±7.8	−217.3±12.1
$G_{sol\text{-}np}$	−17.0±0.2	−16.8±0.1	−16.6±0.1	−16.3±0.2	−15.6±0.1	−16.3±0.2	−15.8±0.3	−16.3±0.2	−17.0±0.1	−17.1±0.2
$G_{sol\text{-}pol,\ GB}$	204.5±16.6	255.3±17.6	291.8±15.5	179.3±9.7	321.9±17.5	243.7±15.7	−116.5±22.2	186.4±13.7	3.3±8.6	122.6±10.9
$G_{sol,\ GB} = G_{sol\text{-}np} + G_{sol\text{-}pol,\ GB}$	187.5±16.6	238.5±17.6	275.2±15.5	163.0±9.6	306.3±17.5	227.3±15.7	−132.3±22.0	170.2±13.6	−13.8±8.6	105.5±10.9
$H_{total,\ GB} = E_{gas} + G_{sol,\ GB}$	−115.7±1.8	−111.3±1.8	−115.3±1.5	−109.0±1.6	−112.9±1.6	−109.9±1.2	−90.8±3.4	−107.9±1.2	−113.4±2.8	−111.7±2.0

*Values given are the energy difference (in kcal/mol) of the Complex(PA_C−$PB1_N$) − PA_C − $PB1_N$. E_{ele}: Coulombic energy. E_{vdw}: van der Waals energy. E_{int}: internal energy. $G_{sol\text{-}np}$: nonpolar solvation free energy. $G_{sol\text{-}pol,GB}$: polar solvation free energy based on the GBSA method. Error given is the SEM. Energies highlighted in blue are more favorable than that of the wild-type complex; energies highlighted in red are less favorable than that of the wild-type complex.
