# Supplementary material for: Host proteostasis modulates influenza evolution
Source: eLife. 2017 Sep 26;6:e28652. doi: 10.7554/eLife.28652 (PMC5614556; doi:10.7554/eLife.28652)
Supplement: Supplementary file 1. [file elife-28652-supp1.docx]

**Supplementary Table 1.** Primer sequences for qPCR and PA sequencing.

| Primer | Sequence |
| --- | --- |
| qPCR: *C. lupus familiaris* *DNAJB1* (fwd) | acatgaactttggccgttcc |
| qPCR: *C. lupus familiaris* *DNAJB1* (rev) | ctctttccatcggggttcag |
| qPCR: *C. lupus familiaris* *HSP70* (fwd) | ggggaggacttcgacaacag |
| qPCR: *C. lupus familiaris* *HSP70* (rev) | ggacgacaaggtcctcttgg |
| qPCR: *C. lupus familiaris* *HSPAA1* (fwd) | tgggttacatggcagcaaag |
| qPCR: *C. lupus familiaris* *HSPAA1* (rev) | agactgaagccggaggacag |
| qPCR: *C. lupus familiaris* *RPLP2* (fwd) | gctacgtcgcctcctacctg |
| qPCR: *C. lupus familiaris* *RPLP2* (rev) | gctcgctgatgaccttgttg |
| qPCR: *Influenza Matrix* (fwd) | agatgagtcttctaaccgaggtcg |
| qPCR: *Influenza Matrix* (rev) | tgcaaaaacatcttcaagtctctg |
| Reverse genetics: Influenza A/Wuhan/1995 *PA* (fwd) | ggctcttggtgaaaacatgg |
| Reverse genetics: Influenza A/Wuhan/1995 *PA* (rev) | aatccttctaattgtggag |
